# Rho-Kinase Inhibitors in the Management of Fuchs Endothelial Corneal Dystrophy: A Review

**DOI:** 10.3390/medicina61050772

**Published:** 2025-04-22

**Authors:** Anđela Jukić, Ana Pupić Bakrač, Biljana Đapic Ivančić, Andrijana Kopić, Ana Meter, Rajka Kasalica Žužul, Josip Pavan, Tomislav Jukić

**Affiliations:** 1 Department of Ophthalmology, University Hospital Dubrava, 10000 Zagreb, Croatia; 2 Department of Neurology, University Hospital Centre Zagreb, 10000 Zagreb, Croatia; 3 Department of Ophthalmology, University Hospital Centre Osijek, 31000 Osijek, Croatia; 4 Department of Ophthalmology, University Hospital Centre Zagreb, 10000 Zagreb, Croatia

**Keywords:** Fuchs endothelial corneal dystrophy, Rho-kinase inhibitors, corneal endothelium, corneal edema, corneal endothelial disorders, corneal endothelial cell regeneration

## Abstract

Fuchs endothelial corneal dystrophy (FECD) is the most common corneal endothelial dystrophy. It is characterized by the progressive loss of corneal endothelial cells (CECs), guttae formation on the Descemet membrane, and corneal edema, leading to visual impairment. Corneal transplantation remains the standard treatment, but it has limitations such as donor shortages, infection risk, and graft rejection. Rho-kinase (ROCK) inhibitors have emerged as a promising pharmacological alternative. These agents promote CEC proliferation, migration, and adhesion while inhibiting apoptosis and enhancing corneal endothelial wound healing. Several studies have demonstrated the efficacy of ROCK inhibitors in improving corneal clarity and endothelial function, particularly when used as an adjunct to Descemet Stripping Only (DSO) surgery. Additionally, they show potential in preventing corneal edema in FECD patients undergoing cataract surgery. The methodology involved a literature search through the PubMed and Medline databases using relevant keywords. Only peer-reviewed articles in English were included, with additional references from selected articles reviewed to ensure comprehensive coverage. ROCK inhibitors offer a novel pharmacological approach to managing FECD. They have shown potential in promoting endothelial cell regeneration and improving corneal functIion. Despite promising results, further research is required to determine ROCK inhibitors’ long-term safety, optimal dosing, and efficacy in surgical and non-surgical FECD patients. Their potential to delay or prevent corneal transplantation represents a significant advancement in FECD management.

## 1. Introduction

FECD is the most prevalent corneal endothelial dystrophy and a leading cause of corneal endothelial dysfunction. It can be classified into two subtypes: early-onset (occurring before the age of 10) and late-onset (more common, occurring after the age of 40). Both subtypes are caused primarily by genetic mutations, but aging and environmental factors (e.g., UV exposure, and trauma) can exacerbate the condition. Early-onset FECD is very rare and is typically associated with severe genetic mutations that disrupt corneal endothelial function very early in life. The pathophysiology of FECD involves mechanisms such as apoptosis, oxidative stress, and abnormal cellular–matrix interactions, which are characterized by excrescences of the Descemet’s membrane (guttae), along with endothelial cell dysfunction and progressive loss of corneal endothelial cells (CECs) [1]. The number of CECs typically declines with age, averaging a decrease of 0.5% per year in healthy individuals [2]. The corneal endothelium consists of a single layer of predominantly hexagonal cells that play a crucial role in maintaining corneal transparency through their pump function and barrier properties. Due to the limited regenerative capacity of CECs, the progression of FECD leads to visual impairment as a result of significant corneal edema. The clinical course of FECD is commonly divided into four stages: Stage 1, characterized by central cornea guttata, often asymptomatic; Stage 2, endothelial decompensation and stromal edema causing visual impairment (particularly upon waking); Stage 3, bullous keratopathy, resulting in discomfort and further vision loss; and Stage 4, marked by subepithelial fibrosis and scarring, sometimes accompanied by corneal neovascularization [3]. Currently, there are no FDA-approved pharmacological treatments available to prevent the progression of FECD. FECD is the leading indication for corneal transplantation in developed countries. The previously utilized surgical technique, penetrating keratoplasty (PKP), has largely been replaced by endothelial keratoplasty (EK) or, more recently, DSO. However, these surgical procedures have limitations, including a shortage of donor corneas, the risk of infection, the side effects of long-term corticosteroid use, and the potential for graft failure and rejection [4]. Consequently, there is a significant demand for pharmacological therapies that could prevent or delay the need for surgery in patients with FECD [5].

Rho-kinase inhibitors (ROCK) are pharmacological agents that inhibit the activity of Rho-associated protein kinase (ROCK). Rho-kinase is a protein kinase modulated by RhoA, a cellular GTPase, and is involved in regulating various cellular processes. In healthy corneal endothelium, basal ROCK activity helps maintain cell shape and adhesion by regulating actomyosin contraction. These effects influence cell morphology and intercellular contacts, contributing to the hexagonal cell shape and barrier function. ROCK also impacts corneal endothelial cell survival; when corneal endothelial cells are subjected to an apoptotic stimulus, ROCK activation leads to myosin-driven contractile force that causes endothelial cells to lose adhesion (detach) and undergo apoptosis [6].

The ROCK pathway plays a crucial role in the pathogenesis of corneal endothelial dysfunction [7]. At the cellular level, the endothelium of eyes with FECD shows evidence of chronic stress and apoptosis, with oxidative stress, mitochondrial dysfunction, and abnormal protein/RNA processing all implicated in its pathogenesis [1]. In this environment, ROCK signaling in corneal endothelial cells appears to be overactivated, which leads to an increase in cellular contractility while reducing the capacity to proliferate or migrate [7]. Furthermore, ROCK signaling is a known facilitator of cytoskeletal and phenotypic transitions, so its upregulation may promote these pathological changes in FECD (e.g., causing cells to become functionally compromised). Importantly, increased ROCK activity may also be involved in the extracellular matrix (ECM) aberrations in FECD. The guttae deposits are composed of collagen and other matrix proteins that endothelial cells in FECD secrete abnormally. Early evidence suggests that ROCK inhibition can reduce this pathological extracellular matrix production in patients with FECD [8].

**Figure 1:**
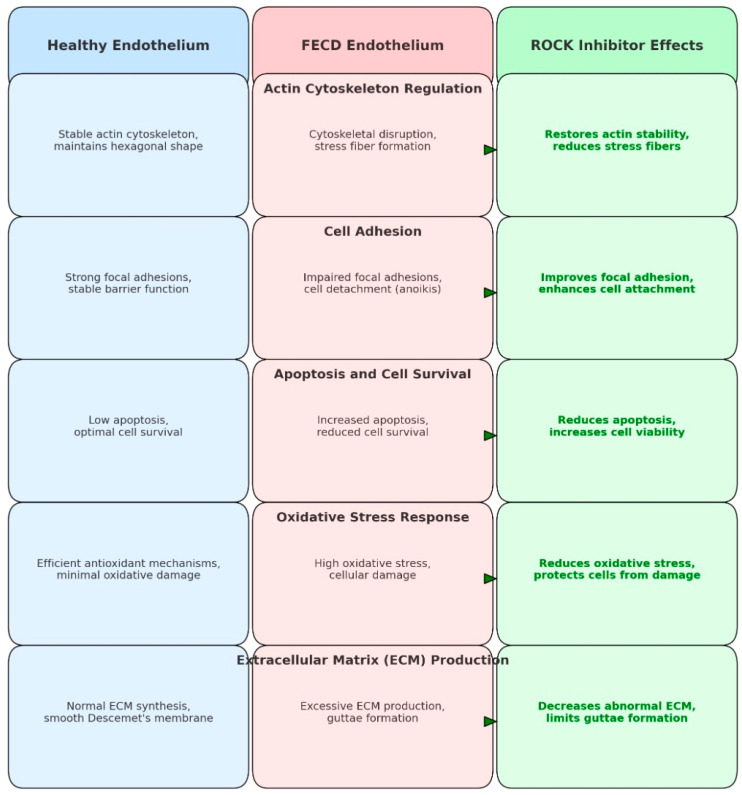
The role of ROCK in healthy cornea (blue outline), Fuchs endothelial corneal dystrophy (FECD, red outline), and effect of ROCK inhibitors (green outline).

ROCK inhibitors affect actin and myosin contractility by increasing cell adhesion, thereby playing a vital role in endothelial barrier function [9]. These inhibitors promote corneal endothelial cell proliferation, inhibit apoptosis, and enhance corneal endothelial wound healing [4,10]. Additionally, it has been suggested that ROCK inhibitors facilitate the migration of healthy endothelial cells from the peripheral cornea to the central and paracentral areas of surgically damaged endothelium [11]. A schematic comparison of the role of Rho-kinase in a healthy corneal endothelium versus in FECD, and the effect of ROCK inhibitors, is shown in Figure 1.

Documented side effects of ROCK inhibitors include conjunctival hemorrhage and hyperemia, blepharitis, allergic conjunctivitis, cornea verticillata, and a novel clinical manifestation known as honeycomb corneal edema (macrocystic or reticular bullous epithelial edema). Honeycomb corneal edema is morphologically distinct from typical microcystic epithelial edema and is characterized by separated edematous cells. Importantly, the development of honeycomb corneal edema does not impact the success or failure of treatment and typically resolves upon discontinuation of the drug [12,13,14,15]. ROCK inhibitor eye drops currently available for commercial use are ripasudil and netarsudil. Ripasudil is a ROCK inhibitor approved in Japan for topical use in glaucoma. It is formulated as an ophthalmic solution (Glanatec™ 0.4%), which is an aqueous eye drop. Another ROCK inhibitor, netarsudil (which also inhibits the norepinephrine transporter), is approved for glaucoma as a 0.02% ophthalmic solution (Rhopressa^®^ and Rocklatan^®^/Roclanda^®^—netarsudil + latanoprost). Y-27632 is typically used in aqueous media for research and has been formulated in preclinical ophthalmic eye drop studies. Y-27632, ripasudil, and netarsudil are ATP-competitive inhibitors of ROCK1 and ROCK2. On a molecular level, ROCK inhibitors suppress actin–myosin contraction and cellular fibrosis pathways, which not only helps endothelial cells spread but may also reduce extracellular deposition on Descemet’s membrane. There is evidence that ROCK inhibition can upregulate genes for junctional and pump proteins, thereby improving endothelial barrier and pump function [16]. Moreover, ROCK inhibitors might reduce the endothelial-to-mesenchymal transition (EMT) of stressed cells, a process implicated in FECD pathology. All these effects contribute to significant improvement in endothelial function [17].

This paper provides a comprehensive review of the current literature on the application of ROCK inhibitors in the treatment of FECD.

## 2. Methodology

The aim of this narrative review was to evaluate the application of ROCK inhibitors in the treatment of FECD. A comprehensive search was performed using two electronic databases: PubMed and Medline. The search strategy involved the use of keywords such as “Rho-kinase inhibitors”, “Fuchs endothelial corneal dystrophy”, “corneal endothelial disorders”, “corneal endothelial cell regeneration”, “corneal edema”, and “corneal endothelium”, as well as their combinations. Medical Subject Headings (MeSH) terms were used where applicable. Boolean operators like “AND” and “OR” were applied to refine the search and ensure the inclusion of the most relevant studies.

All studies published up to March 2025 were considered. Only peer-reviewed articles written in English were eligible for inclusion, provided they specifically addressed the pharmacological use of ROCK inhibitors in the context of FECD. The exclusion criteria included non-English publications, non-peer-reviewed studies, research not involving ROCK inhibitors in FECD, articles focusing on unrelated conditions (such as glaucoma), reviews lacking original data, and studies describing surgical interventions without pharmacological ROCK inhibitor use.

## 3. Results

The systematic search identified a total of 85 potentially relevant articles (63 from PubMed and 22 from Medline). No additional records were found through a manual search of the reference lists or gray literature. After removing 13 duplicate records, 72 articles underwent title and abstract screening. Two independent reviewers performed the screening process, with any disagreements resolved through discussion. Following this initial assessment, 28 articles were selected for full-text review. During the full-text evaluation, 16 articles were excluded for the following reasons: 8 studies lacked a specific focus on FECD, 4 investigated ROCK inhibitors in unrelated conditions such as glaucoma, 3 were review articles without original data, and 1 study focused exclusively on surgical interventions without pharmacological ROCK inhibitor use. The final analysis included 12 studies that met the eligibility criteria. These comprised five randomized controlled trials, four prospective cohort studies, and three case series with sample sizes ranging from 3 to 48 participants in clinical investigations.

Several published studies have demonstrated the efficacy of ROCK inhibitors in preventing and treating corneal edema in patients with FECD. Okumura et al. reported that ROCK inhibitors promote endothelial wound healing and aid in the recovery of central corneal edema after transcorneal freezing in a clinical case series of four FECD patients scheduled for Descemet’s Stripping Automated Endothelial Keratoplasty (DSAEK). Central corneal thickness (CCT) was significantly reduced, and best-corrected visual acuity (BCVA) was significantly improved 6 months after treatment in comparison to pretreatment levels. They suggest that topical ROCK inhibitors improve both the functional and morphological recovery of human CECs [10]. Koizumi et al. reported a case of an FECD patient, scheduled for DSAEK surgery, with completely resolved severe central corneal edema after transcorneal freezing and two weeks of ROCK inhibitor application. Good visual acuity and a clear cornea were maintained for two years after treatment [18].

In an experimental laboratory study, Kruse et al. demonstrated that a topical ROCK inhibitor promotes cell proliferation, adhesion, and migration, while upregulating proteins critical for endothelial pump and barrier function in FECD, not only after acute injury but also prior to surgery. Improvement of endothelial pump and barrier function may delay the indication for surgical intervention [15].

Moloney et al. demonstrated significant improvement in corneal clearance in patients undergoing DSO combined with topical ROCK inhibitors. They compared 23 FECD patients with central corneal edema undergoing DSO and postoperative adjunctive topical ROCK inhibitor therapy with a control group of 9 FECD patients with central corneal edema from a previous study who underwent DSO surgery alone. Corneal clearance was observed in 22 patients of the ROCK inhibitor group. In general, the ROCK inhibitor group achieved clearance more quickly and had significant improvement in BCVA and a decrease in CCT measurements [19].

Macsai et al. compared nine patients who underwent DSO with adjunctive topical ROCK inhibitors to nine patients who underwent DSO alone; the ROCK inhibitor group showed a statistically significant difference in endothelial cell density (ECD) and recovered vision more quickly [20].

Price et al. evaluated the off-label use of topical ROCK inhibitors for treating corneal edema in patients with FECD. They included 29 subjects with corneal edema and FECD, who were randomly assigned to receive either the ROCK inhibitor or placebo. The ROCK inhibitor group showed a significant reduction in CCT and a significant improvement in BCVA compared to the placebo group, three months postoperatively [21].

Similar outcomes were obtained in a study by Lindstrom et al., where patients with FECD and central corneal edema (38 in total) demonstrated significant reductions in CCT and substantial improvements in vision [5].

ROCK inhibitors have shown beneficial effects following acute corneal endothelial cell injury, such as after DSO or cataract surgery, although their efficacy in cases without CEC disruption remains uncertain, as suggested by Kinoshita et al. [1].

Patients affected by FECD represent high-risk cataract surgery cases, 70% of which subsequently need a corneal transplant [22]. The perioperative application of ROCK inhibitors in FECD patients undergoing cataract surgery has been associated with improved ECD and reduced corneal thickness, supporting their role as adjuvant therapy. Antonini et al. presented the beneficial effects of topical ROCK inhibitors used perioperatively in two FECD patients undergoing cataract surgery, and in one case of pseudophakic bullous keratopathy (PBK) while waiting for a scheduled corneal transplantation (DSAEK). After 8 weeks of treatment, the central cornea was clear and BCVA was 20/20. The authors mentioned above suggest that ROCK inhibitors should be considered as adjuvant therapy in cataract surgery for preventing corneal failure in FECD patients and also for improving symptoms in patients scheduled for transplantation surgery [22]. Another recent study explored the use of topical ROCK inhibitors in patients with FECD undergoing cataract surgery. Forty-eight eyes were randomly assigned to receive either a topical ROCK inhibitor or placebo postoperatively, and the study found statistically significant differences in ECD and CCT between the groups [23]. A structured overview of the most relevant studies on using ROCK inhibitors in FECD is shown in Table 1.

## 4. Discussion

Persistent corneal edema resulting from endothelial cell loss or dysfunction is a common sequela in advanced FECD, especially after cataract surgery. The mainstay of treatment is endothelial keratoplasty, but ROCK inhibitors have recently emerged as a pharmacologic approach to promote corneal endothelial healing. Recent results suggest that ROCK inhibitors may facilitate functional repair of corneal endothelial cells in FECD without the need for surgical intervention [19]. As mentioned before, ROCK inhibitors improve cell adhesion, migration, and proliferation, thereby encouraging remaining endothelial cells to cover defects and pump fluid [10,15]. There is evidence that topical ROCK inhibitors can improve corneal endothelial function in FECD, probably by accelerating endothelial cell migration into guttae areas [21]. Also, there is evidence that ROCK inhibitors suppress the secretion of abnormal ECM by cultured human CECs, which could mean that ROCK inhibitors can suppress the development of abnormal Descemet’s membrane deposits (corneal guttae) [24]. Several topical ROCK inhibitors have been studied as a treatment for corneal edema in patients with FECD, with encouraging reports of improved corneal clarity in certain cases [5,10,18,19,21]. Although topical ROCK inhibitors are primarily anti-glaucoma drugs, recent studies have shown their beneficial use for corneal endothelial diseases. There is a growing body of evidence supporting topical ROCK inhibitor efficacy in managing corneal edema, as a therapy or prophylactic measure in patients with low endothelial cell density undergoing cataract surgery. It appears that topical ROCK inhibitors can mitigate surgical trauma to the endothelium, which may help prevent mild edema from progressing to bullous keratopathy. In general, clinical data indicate that these drugs can improve corneal clarity in early to moderate FECD, and they appear most effective when there is a remaining reservoir of healthy endothelial cells in the peripheral cornea [6].

The safety profile of ROCK inhibitors is well documented from large glaucoma trials. The most common side effect is conjunctival hyperemia, which tends to diminish over time. ROCK inhibitors also cause cornea verticillata (vortex keratopathy), but this finding has not been associated with vision loss and typically does not require discontinuation. Serious ocular complications have not been observed, and systemic side effects are rare because systemic exposure is very low [7].

A honeycomb pattern epithelial edema, described as a unique side effect of topical ROCK inhibitors, is relatively uncommon and is reversible. In one report, even without discontinuing the drug, the epitheliopathy self-resolved, suggesting it can be transient [13].

The literature over the past decade indicates that ROCK inhibitors can delay or even avoid the need for corneal transplantation in some cases by recovering endothelial function [25]. However, their limitations must be acknowledged; in advanced FECD with very low endothelial cell counts, endothelial keratoplasty remains the definitive solution [7]. Future clinical trials will further clarify the role of these drugs in the context of postoperative therapy (after DSO or cataract surgery) and as a non-surgical treatment modality for FECD.

In conclusion, topical ROCK inhibitors offer a promising and generally safe new treatment for managing FECD.

## 5. Conclusions and Future Considerations

FECD is a leading cause of corneal endothelial dysfunction and visual impairment, with no FDA-approved pharmacological treatments currently available to prevent its progression. As FECD often leads to the need for corneal transplantation, there is a pressing demand for effective alternative therapies to delay or prevent surgery. ROCK inhibitors have emerged as a promising pharmacological option for managing FECD, with a growing body of evidence supporting their efficacy, especially in patients undergoing cataract surgery.

Additionally, ROCK inhibitors have shown a favorable safety profile, with manageable side effects. Given these promising findings, ROCK inhibitors should be considered a valuable adjunct in the management of FECD, offering a non-surgical alternative that may delay the need for corneal transplantation in affected patients. Further studies are needed to explore the long-term efficacy, safety, and optimal dosing regimens of ROCK inhibitors in both postoperative and non-surgical settings.

## Figures and Tables

**Table 1:** A structured overview of the most relevant studies on using ROCK inhibitors in FECD.

Study	Intervention	Outcome	Reference
Okumura et al.	Topical ROCK inhibitors after transcorneal freezing/clinical case series	Central corneal thickness significantly reduced and BCVA significantly improved	[10]
Koizumi et al.	Topical ROCK inhibitors after transcorneal freezing/clinical case report	Central corneal thickness significantly reduced and BCVA significantly improved	[18]
Kruse et al.	Topical ROCK inhibitors/experimental laboratory study	Promoted endothelial cell proliferation, adhesion, migration, and restoration of endothelial pump and barrier function	[16]
Moloney et al.	DSO + topical ROCK inhibitors vs. DSO alone/clinical study	Faster corneal clearance, central corneal thickness significantly reduced and BCVA significantly improved	[19]
Macsai et al.	DSO + topical ROCK inhibitors vs. DSO alone/clinical study	Faster vision recovery, significantly higher ECD in ROCK inhibitor group 3, 6, and 12 months postoperatively	[20]
Price et al.	Topical ROCK inhibitors vs. placebo for corneal edema treatment/clinical study	Central corneal thickness significantly reduced and BCVA significantly improved 3 months postoperatively	[21]
Lindstrom et al.	Topical ROCK inhibitors for corneal edema treatment/clinical study	Central corneal thickness significantly reduced	[5]
Kinoshita et al.	Topical ROCK inhibitors/brief report	Beneficial effect post-DSO or cataract surgery, efficacy uncertain without CEC disruption	[1]
Antonini et al.	Topical ROCK inhibitors before and after cataract surgery and for treatment of PBK/case report series	ECD and BCVA improvement, corneal clearance	[22]
Keeratidamkerngsakul et al.	Topical ROCK inhibitors vs. placebo after cataract surgery/clinical study	ECD significantly increased	[23]

## Data Availability

Not applicable.
